# Comparing Two Common DNA Extraction Kits for the Characterization of Symbiotic Microbial Communities from Ascidian Tissue

**DOI:** 10.1264/jsme2.ME18031

**Published:** 2018-11-27

**Authors:** James S. Evans, Susanna López-Legentil, Patrick M. Erwin

**Affiliations:** 1 Department of Biology & Marine Biology, and Center for Marine Science, University of North Carolina Wilmington 5600 Marvin K. Moss Lane, Wilmington NC 28409 United States of America

**Keywords:** DNeasy, PowerSoil, bacteria, microbiome, tunicate

## Abstract

Various DNA extraction methods are often used interchangeably for the characterization of microbial communities despite indications that different techniques produce disparate results. The microbiomes of two ascidian species were herein characterized using two common DNA extraction kits, the DNeasy Blood and Tissue Kit (Qiagen) and the PowerSoil DNA Isolation Kit (Mo Bio Laboratories), followed by next-generation (Illumina) sequencing of partial 16S rRNA genes. Significant differences were detected in microbial community diversity and structure between ascidian species, but not between kits, suggesting similar recovery of biological variation and low technical variation between the two extraction methods for ascidian microbiome characterization.

Despite continued technological advances, most microorganisms remain resistant to laboratory culturing techniques, which necessitates other means of microbial identification and detection. Accordingly, recent microbiome research has standardized the use of high-throughput DNA sequencing technologies in the characterization of entire microbial communities from different hosts and habitats (10). As the utilization of next-generation DNA sequencing continues to expand, there has been a corresponding demand for the development of high-yield, high-quality DNA extraction techniques. A wide variety of pre-assembled DNA extraction and isolation kits are now commercially available and are often used interchangeably, even to answer similar scientific questions. This results in comparisons across studies that have obtained DNA in disparate manners, raising questions regarding the introduction of technical variation that may confound these comparisons. For example, previous studies have demonstrated that different extraction techniques yield highly variable data, with DNA qualities and yields fluctuating in response to the kit type (1, 11, 19, 20, 26) or target taxa (11, 20), which may also have an impact on analyses of symbiotic microbial communities (11, 12, 26).

Ascidians (subphylum Tunicata) represent a highly diverse lineage of the phylum Chordata, with solitary and colonial types and approximately 3,000 described species (24). Previous studies revealed that ascidians host diverse and abundant microbial communities (3, 6, 8), which appear to maintain a constant structure across broad spatial (3) and temporal scales (15, 17). These microbial communities have been reported within the inner portion of the ascidian tunic, a cellulosic coating that surrounds and protects the animal and is separated from the branchial sac utilized in filter feeding and the digestive system (6, 7). However, broader conclusions regarding the host-specificity and stability of ascidian-associated microbial communities require meta-analyses based on cross-study data, for which the use of different DNA extraction methods may confound comparisons. In the present study, we investigated whether two commonly utilized DNA extraction kits, the DNeasy Blood and Tissue Kit (Qiagen, Hilden, Germany) and the PowerSoil DNA Isolation Kit (Mo Bio Laboratories, Hilden, Germany), produce similar characterizations of ascidian microbiomes. Both extraction kits have been widely utilized to characterize symbiotic microbial communities in ascidians (2, 8, 14–16, 23), with the latter kit (PowerSoil) being utilized in the standardized DNA extraction protocol established by the Earth Microbiome Project (10).

In the present study, two colonial ascidian species common to the North Carolina coastline were investigated: *Clavelina oblonga* (Herdman, 1880; Fig. S1a) and *Polyandrocarpa anguinea* (Sluiter, 1898; Fig. S1b). Replicates of *C. oblonga* (*n*=5) were collected in July 2014 from the Olde Towne Yacht Club in Beaufort North Carolina (NC), USA (34°42′56″N 76°40′44″W). Replicates of *P. anguinea* (*n*=5) were collected in September 2015 from Wrightsville Beach Marina (34°12′59.6″N 77°48′45.7″W), in Wrightsville Beach NC, USA. All samples were obtained at a depth <1 m, and sampled at least 5 m apart to reduce the likelihood of sampling clones. Replicate ascidian samples were collected in sterile Whirl-Pak bags containing ambient seawater and processed within 1 h. A portion of each ascidian colony was rinsed with filtered seawater and stored in absolute ethanol at −20°C for molecular analyses. Ethanol-preserved ascidian samples were then dissected under a stereomicroscope and a portion of the inner tunic (*ca.* 3 mm^3^) was extracted for microbial symbiont characterization. Tunic tissue is comprised of host cells and extracellular symbionts in a polysaccharide (cellulose-like) matrix, with the inner tunic indicating tissue not in contact with either the surrounding seawater or zooids (thereby reducing contamination from transient or prey microbes). To compare the efficacy of the two DNA extraction kits, tunic tissue from each replicate was processed separately using the DNeasy Blood and Tissue Kit (Qiagen, hereafter “DNeasy”) and PowerSoil DNA Isolation Kit (Mo Bio Laboratories, hereafter “PowerSoil”) following the manufacturers’ protocols. DNA yields from tunic extractions were quantified on a NanoDrop One spectrophotometer (Thermo Scientific, Waltham, MA, USA). The PowerSoil kit obtained average DNA yields of 82±44 ng (±1 SE) and 68±24 ng, with average a260/a280 ratios of 1.12±0.18 and 1.61±1.16, for *C. oblonga* and *P. anguinea*, respectively. In comparison, the DNeasy kit obtained average yields of 945±171 ng and 6,939±1,820 ng, with average a260/a280 ratios of 2.03±0.12 and 1.89±0.02, for *C. oblonga* and *P. anguinea*, respectively. DNA extracts were used as templates for the PCR amplification and sequencing of *ca.* 300-bp fragments (V4 region) of 16S ribosomal RNA (rRNA) genes. Amplification was performed using the universal bacterial/archaeal primers 515f and 806r (4) to confirm the viability of DNA extraction. Viable DNA extracts were sent to Molecular Research LP (Shallowater, TX, USA) for amplification, library construction, and multiplexed sequencing on an Illumina MiSeq platform (Illumina, San Diego, CA, USA). Raw sequence data were stored in the Sequence Read Archive of NCBI (accession no. SRP125054). Sequence data for *P. anguinea* sequences extracted with DNeasy were published previously (8) and retrieved from NCBI (accession no. SRP106072).

Raw sequences were processed in the mothur software package (21) following a modified version of the Illumina MiSeq SOP pipeline (13). Briefly, raw sequences were quality-filtered and aligned to the Silva reference database (SSU Ref NR 99 v128), and then classified with a naive Bayesian classifier and bootstrap algorithm for confidence scoring (25), constructed based on the improved Greengenes taxonomy (18). Non-target sequences and singletons were removed from the data set, and putative chimeric sequences were removed via self-reference searching with UChime (5). Using the OptiClust clustering algorithm in mothur, high-quality sequences were assigned to operational taxonomic units (OTUs) based on 97% sequence similarity, and the taxonomic classification of each OTU was selected via a majority consensus (22). Sampling depths (*i.e.*, the number of sequence reads) among the different replicates were standardized by subsampling to the lowest read count (*n*=20,007) from the final shared file, and all subsequent data analyses utilized these subsampled data sets.

To compare microbial community diversity across ascidian species and extraction methods, alpha diversity metrics for OTU richness and evenness were calculated in mothur, including observed OTU richness (S), expected OTU richness (Chao1), the Simpson evenness index (E1/D), inverse Simpson index (D), and Shannon-Weaver diversity index (H’). Analyses of variance (ANOVA) were used to statistically compare diversity indices for the factors source (*C. oblonga* vs. *P. anguinea*), kit (DNeasy vs. PowerSoil), and an interaction term. Tukey’s honest significance difference tests were performed for multiple pairwise post hoc comparisons of means.

To compare microbial community structures across ascidian species and extraction methods, Bray-Curtis similarity (BCS) matrices were calculated based on OTU relative abundance in PRIMER (version 7.0.13) and visualized in non-metric multidimensional scaling plots. Permutational multivariate analyses of variance (PERMANOVA) were used to statistically compare the structure of microbial communities for the factors source, kit, and an interaction term. Permutational multivariate analyses of dispersion (PERMDISP) were performed to verify that significant PERMANOVA results stemmed from structural differences, and not the unequal dispersion of variability among groups. To investigate host and kit effects on dominant vs. rare members of the microbiome, sequence data were divided into abundant and rare-OTU partitions using a 0.1% relative abundance threshold (9), resulting in a cut-off value of 20 sequences (“abundant” OTUs contained >20 sequences, “rare” OTUs contained ≤20 sequences). BCS matrices were created for each data partition and PERMANOVA was used to test for differences in the structure of abundant and rare members of the microbiome, as described above.

A total of 5,978 distinct microbial OTUs were detected. Using the DNeasy kit, 3,097 and 3,080 OTUs were obtained from *C. oblonga* and *P. anguinea*, respectively. Using the PowerSoil kit, 2,438 and 2,716 OTUs were obtained from *C. oblonga* and *P. anguinea*, respectively. Between the kits, 2,565 OTUs were obtained using both kit types, while 2,077 and 1,336 OTUs were unique to the DNeasy and PowerSoil kits, respectively. Within each species, 1,537 OTUs were shared across both extraction methods for *C. oblonga* and 1,664 OTUs for *P. anguinea*. A total of 882 OTUs were detected in both hosts using both kits.

Ascidian microbial communities exhibited high diversity, as previously reported for other species (3, 7, 8), with detected OTUs spanning 42 bacterial and three archaeal phyla. Clear interspecific differences and low intraspecific variation in dominant microbial taxa were observed (Fig. 1a), which were consistent with previous findings (3, 7, 8). Intraspecific variation across kits reflected slight differences in the relative abundance of some taxa (Fig. 1a). Using the DNeasy kit, *C. oblonga* symbionts were found to include 40 microbial phyla, with the highest relative abundances of *Alphaproteobacteria* (30%), *Gammaproteobacteria* (20%), *Deltaproteobacteria* (15%), and *Epsilonproteobacteria* (13%; Fig. 1b). Using the PowerSoil kit, *C. oblonga* symbionts spanned 37 microbial phyla, and were again dominated by *Alphaproteobacteria* (36%), *Gammaproteobacteria* (26%), *Epsilonproteobacteria* (13%), and *Deltaproteobacteria* (6%; Fig. 1c). Using the DNeasy kit, *P. anguinea* microbial communities were found to include 38 microbial phyla, and were dominated by *Alphaproteobacteria* (66%), *Crenarchaeota* (13%), *Gammaproteobacteria* (7%), and *Planctomycetes* (4%; Fig. 1d). Using the PowerSoil kit, *P. anguinea* microbial communities were found to include 40 microbial phyla, with the highest relative abundances of *Alphaproteobacteria* (66%), *Gammaproteobacteria* (12%), *Planctomycetes* (5%), and *Crenarchaeota* (4%; Fig. 1e).

Alpha diversity metrics further indicated a minimal effect of the extraction method on ascidian microbial community diversity. Of the five diversity metrics tested, only the Chao 1 index exhibited a significant difference between DNA extraction kits (*P*=0.03, Table S1). In contrast, host species (*C. oblonga* or *P. anguinea*) was found to have a significant effect (*P*<0.05) on three diversity indices: Simpson, inverse Simpson, and Shannon-Weaver (Table S1). In all three cases, *C. oblonga* exhibited greater microbial community diversity than *P. anguinea*. No significant interactive effect was detected between the host species and kit for any of the diversity indices (Table S1).

Extraction method exhibited an even weaker impact on the observed ascidian microbial community structure. Microbial communities clustered strongly in response to the ascidian source (Fig. 2), with significant differences detected between sources (*C. oblonga* vs. *P. anguinea*, PERMANOVA, *P*=0.001), but not kits (DNeasy vs. PowerSoil, PERMANOVA, *P*=0.346), and no significant interactive effect between factors (PERMANOVA, *P*=0.448, Table S2). Source contributed to 67.3% of the observed variation in the microbial community structure, while kit was responsible for only 0.31% of this variability. Consequently, SIMPER analyses revealed a greater average dissimilarity between species within each kit (DNeasy=88.17%, PowerSoil=88.18%) than within species between the kits (*C. oblonga*=51.99%, *P. anguinea*=46.44%). No significant difference in dispersion was detected across the two species (PERMDISP, *P*=0.318) or across the extraction kits (PERMDISP, *P*=0.532, Table S2).

To clarify whether differences in OTU relative abundance drive the variations observed in microbial community structure, microbial community data were partitioned into abundant (>0.1% relative abundance) and rare (≤0.1%) components. Partitioning resulted in 852 abundant OTUs that represented 95.2% of all sequences, and 5,126 rare OTUs that accounted for the remaining 4.8% of all sequences, post singleton removal. Abundant and rare data partitions both followed similar trends as the overall data set, indicating significant differences in microbial community structure among host species (PERMANOVA, *P*=0.001), no significant differences between extraction methods (PERMANOVA, *P*≥0.288), and no significant interactive effect between the two factors (PERMANOVA, *P*≥0.424, Table S2). No significant differences were observed in dispersion for either the abundant or rare data partitions across sources (PERMDISP, *P*=0.303 and *P*=0.717, respectively), or across extraction kits for the abundant data partition (PERMDISP, *P*=0.561). However, a significant difference in dispersion across the kit types was detected for the rare data partition (PERMDISP, *P*=0.008, Table S2).

While previous studies reported that the characterization of microbial symbionts may be affected by the choice of extraction method (11, 12, 26), the magnitude of this effect is variable and often less than the biological parameters under study. For example, human microbiome studies have shown greater variability between individuals than among extraction methods (12, 26), with similar results observed in other mammalian gut microbiome studies (11). The technical variation introduced by different extraction methods is often lower than biological variation, thereby leading to greater concern when the effects of biological parameters are subtle. Furthermore, variation may be observed among extraction methods within some host taxa and not others (11). In the present study, no significant differences in extraction kits were observed for beta diversity metrics and only one of the five alpha diversity metrics significantly differed across kits. In contrast, a clear biological signal of host-specificity was observed across both kits and data partitions (abundant and rare microbiome members). While some intra-individual variation was observed, this variation was often consistent across kits (*e.g.* individual 4 of *C. oblonga*) and likely reflects true differences between individuals. Collectively, these results revealed comparable recovery of biological variation and low technical variation across common DNA extraction methods for ascidian microbiome characterization, demonstrating that either approach may be utilized to reliably describe ascidian symbiotic microbial communities.

## Supplementary Material



## Figures and Tables

**Fig. 1:**
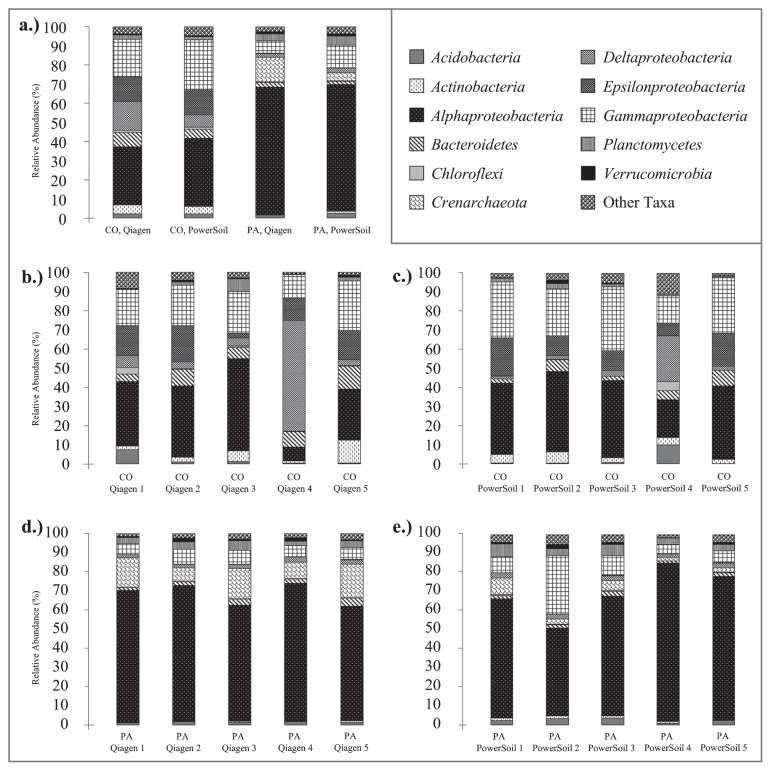
Microbial community composition averaged by source and kit (a), and for each replicate sample within each source: *Clavelina oblonga*, DNeasy (b), *C. oblonga*, PowerSoil (c), *Polyandrocarpa anguinea*, DNeasy (d), and *P. anguinea*, PowerSoil (e). Phylum-level classifications are shown, except for *Proteobacteria*, which were divided into four classes: *Alpha*-, *Gamma*-, *Delta*-, and *Epsilonproteobacteria*. Each library represents 20,007 sequence reads.

**Fig. 2:**
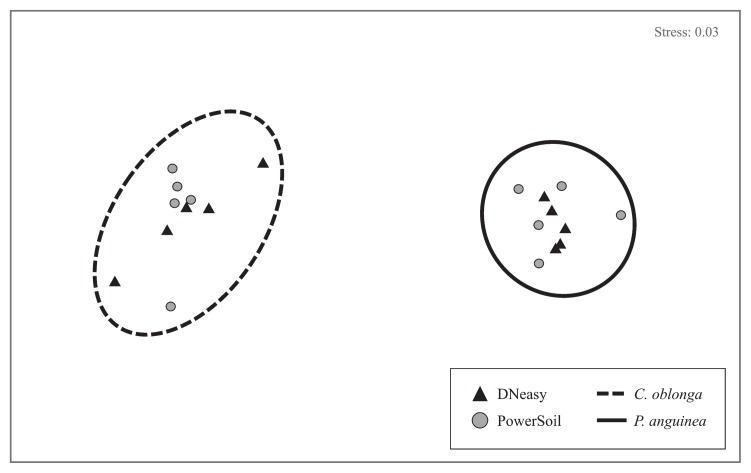
Non-metric multi-dimensional scaling plot based on the Bray-Curtis similarity of microbial communities obtained using DNeasy (black triangles) and PowerSoil (gray circles) kits. Circles encompass samples clustering at >30% similarity and correspond to replicates of *Clavelina oblonga* (dashed line) and *Polyandrocarpa anguinea* (solid line), indicating a high degree of host-specificity and low technical variation across DNA extraction methods.
