# Supplementary material for: Comparing Two Common DNA Extraction Kits for the Characterization of Symbiotic Microbial Communities from Ascidian Tissue
Source: Microbes Environ. 2018 Nov 27;33(4):435–9. doi: 10.1264/jsme2.ME18031 (PMC6308000; doi:10.1264/jsme2.ME18031)
Supplement: Supplementary file 1 [file 33_435_s1.pdf]

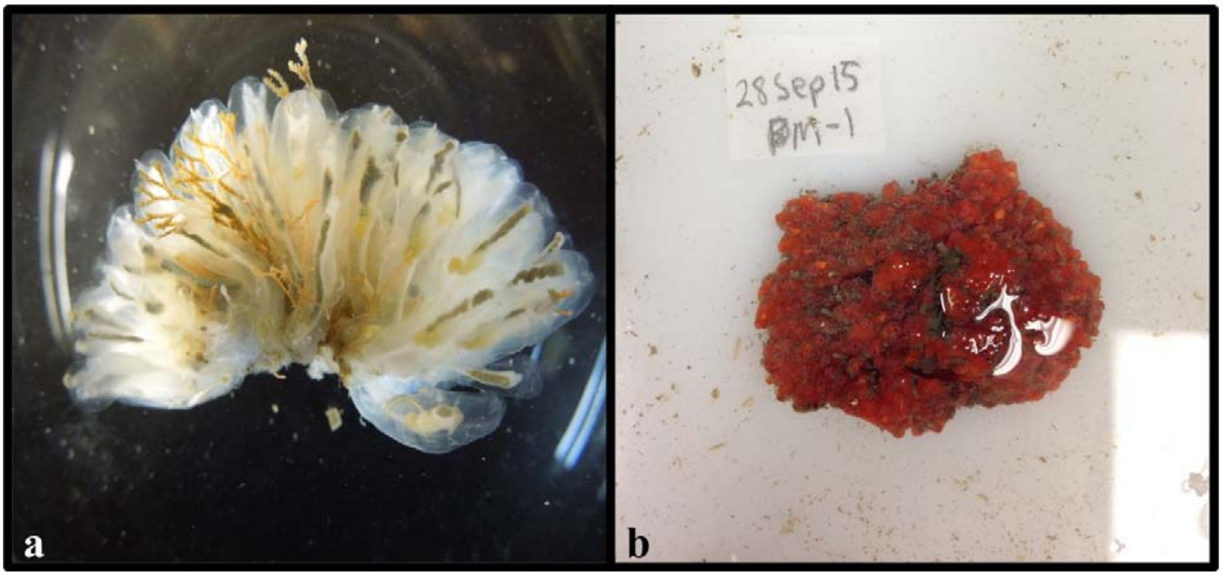

Fig. S1. Representative photographs of the colonial ascidians *Clavelina oblonga* (a) and *Polyandrocarpa anguinea* (b).

Table S1. Diversity metrics for microbial communities associated with *Clavelina oblonga* and *Polyandrocarpa anguinea*, extracted using the DNeasy Blood and Tissue kit (Qiagen) and the PowerSoil DNA Isolation kit (Mo Bio Laboratories, Inc.). Average values (±1 SE) are shown, with different superscript letters denoting significantly different means among sources. *S*: observed richness, *Chao 1*: expected richness, *E1/D*: Simpson Evenness, *D*: Inverse Simpson, *H'*: Shannon Weaver.

**Source**	**S**	**Chao 1**	**E1/D**	**D**	**H'**
C. oblonga	1026.1 ± 95.2^{A}	1903.0 ± 163.6^{A}	0.015 ± 0.003^{A}	17.12 ± 4.751^{A}	4.012 ± 0.241^{A}
P. anguinea	1100.7 ± 86.9^{A}	1924.7 ± 135.3^{A}	0.007 ± 0.002^{B}	6.724 ± 0.681^{B}	3.310 ± 0.119^{B}

**Kit**	**S**	**Chao 1**	**E1/D**	**D**	**H'**
DNeasy	1186.3 ± 81.41^{A}	2144.9 ± 134.4^{A}	0.011 ± 0.003^{A}	13.92 ± 5.156^{A}	3.640 ± 0.281^{A}
PowerSoil	940.50 ± 83.33^{A}	1682.8 ± 123.2^{B}	0.012 ± 0.002^{A}	9.923 ± 1.251^{A}	3.682 ± 0.144^{A}

**Source*Kit**	**S**	**Chao 1**	**E1/D**	**D**	**H'**
C. oblonga (DNeasy)	1160.0 ± 166.3^{A}	2159.6 ± 279.4^{A}	0.016 ± 0.005^{A}	22.14 ± 9.252^{A}	4.104 ± 0.488^{A}
C. oblonga (PowerSoil)	892.20 ± 64.56^{A}	1646.4 ± 97.46^{A}	0.014 ± 0.002^{A}	12.10 ± 1.836^{A}	3.921 ± 0.138^{A}
P. anguinea (DNeasy)	1212.6 ± 42.61^{A}	2130.3 ± 56.25^{A}	0.005 ± 0.000^{A}	5.701 ± 0.504^{A}	3.177 ± 0.097^{A}
P. anguinea (PowerSoil)	988.80 ± 161.0^{A}	1719.2 ± 241.1^{A}	0.009 ± 0.003^{A}	7.748 ± 1.144^{A}	3.442 ± 0.214^{A}

Table S2. Statistical analyses of microbial community structure (PERMANOVA) and dispersion (PERMDISP) in ascidians across the factors source (*Clavelina oblonga* vs. *Polyandrocarpa anguinea*), kit (DNeasy vs. PowerSoil) and an interaction term (source x kit). Comparisons of overall, abundant, and rare OTU data partition levels are shown.

	**Overall**				**Abundant**				**Rare**			
	PERMANOVA		PERMDISP		PERMANOVA		PERMDISP		PERMANOVA		PERMDISP	
**Factor**	*Pseudo-F*	*p*	*t*	*p*	*Pseudo-F*	*p*	*t*	*p*	*Pseudo-F*	*p*	*t*	*p*
Source	20.72	**0.001**	1.219	0.318	22.58	**0.001**	1.267	0.303	2.042	**0.001**	0.461	0.717
Kit	0.909	0.346	0.558	0.532	0.886	0.383	0.511	0.561	1.034	0.288	3.373	**0.008**
Source x Kit	0.836	0.448	n.a.	n.a.	0.831	0.424	n.a.	n.a.	0.898	0.844	n.a.	n.a.
